# Supplementary material for: Addressing Combative Behaviour in Spanish Bulls by Measuring Hormonal Indicators
Source: Vet Sci. 2024 Apr 22;11(4):182. doi: 10.3390/vetsci11040182 (PMC11053816; doi:10.3390/vetsci11040182)
Supplement: Supplementary file 1 [file vetsci-11-00182-s001.zip › vetsci-2822367-supplementary.pdf]

Supplementary file material and methoids:

Direct observational method:

The template was divided into main three parts. The first part referred to the aggressive actions developed by the bull in each specific moment of the bullfight (exit to the arena, third of sticks, third of skewers and third of crutch). Each part was, in turn, subdivided into different sections that specified the actions that indicate aggressiveness in the fighting bull. The observers scored these sections in a range from 1 to 5, with 1 being the partial score of the animal that does not present any of the aggressive actions and 5 the partial score of the animal that has developed the four aggressive actions specified in each part. Thus, for each aggressive action developed by the bull, one point would be added to each partial score. The average of the three partial scores, corresponding to the three parts of the bullfight, was calculated, which resulted in a preliminary score.

The second part of the template referred to the "general impression" that the spectator had of the bull behaviour and served to modulate the preliminary mark obtained in the first part. For each of the points of this section, 0.25 points were added to the preliminary score, with 1 being the maximum mark to be added. In any case, this section helped to classify more accurately the aggressiveness of the bull, so that in the cases of clearly aggressive bulls, the maximum final score would never exceed the value of five, and therefore, it would not be necessary to rate this second section.

The third part, called observations, consisted of the collection of significant observations to assign an aggressiveness score to each animal. The aggressive actions described in the two previous sections indicated whether aggressiveness had been acquired. In the case of acquired aggressiveness, we differentiated the acts carried out by the bullfighters that could provoke this acquired aggressive response, such as crashing the bulls against the racks, bad application of the third of sticks and skewer, or even submitting a bull lacking in strength to the provocation of the stimuli. Finally, acquired aggressiveness refers to all the actions undertaken by the bulls when lunged and which do not consummate the act due to physical impediment or an inadequate handling that can frustrate the action of the bulls, thus increasing aggressiveness.

The observational method described was rigorously applied using three trained observers to evaluate the acts of aggressive behaviour of the bulls during the bullfight, thus minimizing the intra-observer error (given the complication of assigning an objective value to a specific behaviour). The aggressive behaviour score corresponding to each animal was assigned during the bullfight.

This allowed us to determine an aggressiveness score for their behaviour in a more objective way since this template only recorded the aggressive actions of the animal:

1: Very slightly aggressive

2: Not very aggressive

3: Combative

4: Aggressive

5: Very aggressive

Certain behavioural actions were considered indicators of aggressiveness during the fight, such as:

- Repeating the onslaught with greed or vehemence.

- Hesitating in the attack and, when attacking, to shorten the predictable trajectory, to look for the bullfighter.

- Threatening and hesitating very often when presented with stimuli.

- Nodding, attacking with face up and trying to defend against the deceptions quickly and unexpectedly, and trying to attack its opponent (defeat).

- Kicking, vocalising in different versions and attacking or not to the deceptions.

**Date:**	**Arena:**		
**Number:**	**Cattle breeding:** **Encaste:**		
**Age:**	**Bull ID:**		
			**PARTIAL SCORE**
**EXIT TO THE ARENA**	**Doubt in the onslaught**		
	**Repeat the charge with greed**		
	**Cut the onslaught and look for the bullfighter**		
	**Defeat in deception**		**(1–5)**
**THIRD OF STICKS**	**The bull greedily starts the horse**		
	**Nods wanting to remove the stick**		
	**Looks for more and new places to defeat**		
	**Does not come loose**		**(1–5)**
**THIRD OF SKEWERS**	**Cuts the trajectory of the stickerman**		
	**Face up**		
	**Chases the stickerman**		
	**It hurts**		**(1–5)**
**THIRD OF CRUTCH**	**Repeat with greed**		
	**Doubt in the onslaught**		
	**Cuts back on the onslaught**		
	**Attacks defeating in the deceptions**		**(1–5)**
			**AVERAGE SCORE**
**GENERAL IMPRESSION**	**Mobility**		
	**Discover the cheated**		**(0–1)**

	**Transmission of danger**		
	**Kicks, digs, vocalizes during the fight**		
**OBSERVATIONS**	**Force:**		
	**Lidia:**		
	**Others**		**FINAL SCORE**

Table S1. Template for observational data collection during the bullfight.
